# High Mechanic Enhancement of Chopped Carbon Fiber Reinforced-Low-Density Unsaturated Polyester Resin Composite at Low Preparation Temperature with Facile Polymerization

**DOI:** 10.3390/ma14154273

**Published:** 2021-07-30

**Authors:** Jian Zhang, Xiaojun Wang, Xinjun Fu

**Affiliations:** Department of Composite Materials, College of Materials Science and Engineering, Nanjing Tech University, Nanjing 211800, China; 201861203151@njtech.edu.cn (J.Z.); 201961203156@njtech.edu.cn (X.F.)

**Keywords:** chopped carbon fiber, low-density unsaturated polyester resin, synergistic effect, facile polymerization, high mechanic enhancement

## Abstract

Chopped carbon fiber-reinforced low-density unsaturated polyester resin (CCFR-LDUPR) composite materials with light weight and high mechanical properties were prepared at low temperature and under the synergistic action of methyl ethyl ketone peroxide (MEKP-II) and cobalt naphthenate. Optimal preparation conditions were obtained through an orthogonal experiment, which were preparation temperature at 58.0 °C, 2.00 parts per hundred of resin (phr) of NH_4_HCO_3_, 4.00 phr of chopped carbon fibers (CCFs) in a length of 6.0 mm, 1.25 phr of initiator and 0.08 phr of cobalt naphthenate. CCFR-LDUPR composite sample presented its optimal properties for which the density (*ρ*) was 0.58 ± 0.02 g·cm^−3^ and the specific compressive strength (*P_s_*) was 53.56 ± 0.83 MPa·g^−1^·cm^3^, which is 38.9% higher than that of chopped glass fiber-reinforced low-density unsaturated polyester resin (CGFR-LDUPR) composite materials. Synergistic effects of initiator and accelerator accelerated the specific polymerization of resin in facile preparation at low temperature. Unique “dimples”, “plate microstructure” and “surface defect” fabricated the specific microstructure of the matrix of CCFR-LDUPR composite samples, which was different from that of cured unsaturated polyester resin (UPR) with “body defect” or that of CGFR-LDUPR with coexistence of “surface defect” and “body defect”.

## 1. Introduction

Carbon fiber-reinforced plastic is a typical advanced composite material with light weight and high mechanical performance [1,2]. Among them, chopped carbon fiber-reinforced thermosetting resin-based composites are already indispensable in the automotive industry, aviation industry, sports equipment, wind power, military industry and other fields due to their light weight, high strength, easy molding, high temperature resistance, and corrosion resistance [3]. As a novel light-weight material, low-density unsaturated polyester resin composites were prepared by foaming methods. It combined with chopped glass fibers or fillers to form composites, including chopped glass fiber-reinforced low-density unsaturated polyester resin (CGFR-LDUPR) [4,5]. In the future, researches of chopped carbon fiber-reinforced low-density unsaturated polyester resin (CCFR-LDUPR) composites will be the highlight of chopped carbon fiber-reinforced thermosetting resin-based composites or low-density unsaturated polyester resin based composites.

Applications of chopped carbon fiber were mostly used in epoxy resin to prepare composite materials. Chai et al. [6] improved the flame retardancy, thermal stability, and mechanical properties of the epoxy resin composite, utilizing a solution mixing method for chopped carbon fibers-epoxy resin composite by an addition of 0.7 wt% chopped carbon fibers. An external alternating current electric field was applied to orient the chopped carbon fibers in its epoxy resin composite and to improve the fracture toughness of the composite [7]. Chandran et al. [8] pointed out that basalt fiber could improve mechanical properties and wear properties of chopped carbon fibers-epoxy resin composite. Srivastava et al. [9] discussed the effect of multi-walled carbon nanotubes on the mechanical and electrical properties of chopped carbon fibers-epoxy resin composite. Chopped carbon fibers were utilized in other composites of thermosetting resin matrix. Ahmadijokani et al. [10] reported the effects of chopped carbon fibers on the thermal, mechanical and tribological properties of phenolic-based brake friction materials. All of the researches presented above were assigned to the performance of chopped carbon fibers in solid composites of thermosetting resin matrix, and had not been conducted in low-density composite materials. Recently, chopped glass fiber in a content of 20.0 parts per hundred of resin (phr) was first applied to low-density unsaturated polyester resin by Guo et al. [4] at 76 °C, with the highest specific compressive strength for chopped glass fiber reinforced low-density unsaturated polyester resin.

Different to the above applications of carbon fiber in epoxy resin, and different from previous preparation of low-density unsaturated polyester resin composites at moderate temperature [4,5], carbon fiber was used in unsaturated polyester resin and CCFR-LDUPR composite was prepared at a low temperature from 52.0 to 60.0 °C, NH_4_HCO_3_, a foaming agent with a wide decomposition temperature range, was used in the study. Synergistic effects at lower temperature were realized in this study in the presence of methyl ethyl ketone peroxide (MEKP-II) and an accelerator of cobalt naphthenate. The polymerization of resin in this facile preparation at low temperature was characterized by differential scanning calorimetry (DSC) technology. The microstructure changes of resin, chopped carbon fibers, and bubbles during the facile preparation, as well as the interaction between chopped carbon fibers and unsaturated polyester resin (UPR) were explored by optical microscope observation and scanning electron microscope (SEM) technology. The composite of chopped carbon fibers and low-density unsaturated polyester resin will extend the application of low-density composite materials. The facile preparation of CCFR-LDUPR composite at a low temperature presented in this work will promote an advanced preparation of low-density composites.

## 2. Experimental

### 2.1. Materials

Unsaturated polyester resin was XP810-901 bisphenol A vinyl resin (in a solid content of 56–59 wt%) with styrene as the solvent (made by AOC Aliancys Resin Co., Ltd., Nanjing, China). At 23 ± 1 °C, the viscosity was 2000–2300 MPa·s. The acid value was 22–25 mg·KOH·g^−1^, and the number-average molecular weight was 2017 ± 15 g·mol^−1^.

The carbon fiber was HF10-3K-E PAN-based, which was produced by Jiangsu Hengshen Co., Ltd., Zhenjiang, China. The length of the fiber after it was chopped was 2, 4, 6, 8 and 10 mm. The linear density was 198 g/km, and the diameter of fiber was 7 μm.

The glass fiber was ECR13-300D-608 from Taishan Glass Fiber Co., Ltd. (Taian, China). The length of chopped glass fiber was 6.0 mm. The linear density was 300 g/km, and the diameter of fiber was 13 μm.

The foaming agent NH_4_HCO_3_ came from Shanghai No. 4 Reagent & H.V. Chem. Co., Ltd., Shanghai, China, which contained more than 99 wt% NH_4_HCO_3_.

Methyl ethyl ketone peroxide (MEKP-II) an initiator was the product from Luoyang Shuangyue Curing Agent Co., Ltd., (Luoyang, China) with a peroxide content of 33% and an active oxygen content of 8.9%. 

The accelerator was NL-49P cobalt naphthenate, which was produced by Akzo Nobel Co., Ltd. (Tianjin, China), contained 1% by mass of Co. 

The release agent was PMR-EZ made by Chem-Trend Chemicals Co., Ltd, (Shanghai, China) and the content was more than 99 wt%. 

### 2.2. Preparation of CCFR-LDUPR Specimens

According to Reference [4], CCFR-LDUPR composite samples were prepared at a certain temperature in a formula listed in Table 1. The content in Table 1 is the mass part corresponding to 100 grammes resin (phr). 

Value A was the amount of the foaming agent within 3.00 phr according to Reference [11]. The values of B, C, and D were respectively obtained from preliminary experiments of the study. Chopped carbon fibers were added into the unsaturated polyester resin glue utilizing a stirrer (SJB-400, Jintan Hengfeng Instrument Factory, Changzhou, China) at a speed of 120 r/min. The mixture was stirred for 5 min at this speed until chopped carbon fibers were evenly distributed homogeneously in resin glue. Later, the mixture was added into a mold uniformly, and then cured at a certain temperature for 1.5 h. After that, the cured specimen was cooled down to room temperature and then demolded. In a parallel experiment, five replicated specimens were tested for each formulation.

### 2.3. Methods

#### 2.3.1. Microscope Observation

Distribution of chopped carbon fibers and bubbles in resin glue was observed by an auto-fine-tune portable microscope (A005+, Shenzhen D&F Co., Shenzhen, China) at magnifications of up to 500×.

#### 2.3.2. Viscosity Test

According to the standard of ISO 2555:2018, viscosity of unsaturated polyester resin glue with different contents and lengths of chopped carbon fibers was measured by a digital rotational viscometer (NDJ-79A, Shanghai Changji Geological Instrument Co., Ltd., Shanghai, China), with a rotational speed of 750 rpm, a sensor accuracy of 2% full scales, and with a relative humidity lower than 80%.

#### 2.3.3. Gel Time Test

According to the standard of ISO 2535:2001, gel time of unsaturated polyester resin at different temperatures was measured by a gel time meter (GT-2, Lin’an Fengyuan Electronics Co., Ltd., Hangzhou, China), with a temperature accuracy of ±0.1 °C and a working temperature no more than 230 °C.

#### 2.3.4. Mechanical Properties Test

Samples were cut into cylinders with a bottom diameter of 60 ± 1 mm and a height of 50 ± 1 mm. The density of the samples was tested according to the standard ISO 84:2006. According to the standard of ISO 844:2014, the compressive strength of rigid foam plastics was measured by an electronic universal tensile testing machine (WDW3100, produced by Changchun Xinke Testing Machine Co., Ltd., Changchun, China), with a maximum pressure of 100 kN, a force accuracy of ±0.5%, and a test speed of 0.05–500 mm·min^−1^). The test speed applied was 5 mm·min^−1^, ambient temperature was 23 ± 2 °C, and relative humidity was 50 ± 5%.

#### 2.3.5. Non-Isothermal Differential Scanning Calorimetry (DSC) Testing

Non-isothermal DSC tests of sample curing were examined using Netzsch DSC204 (NETZSCH, Selb, Germany). The test was carried out using approximately 10 mg for a sample, which was sealed in an aluminum crucible. The sample was heated from 25 to 200 °C at a rate of 10 °C·min^−1^, and then cooled from 200 to 25 °C at a rate of 30 °C·min^−1^. The flow rate of nitrogen to the balance area was 60 mL·min^−1^, and the sample was swept at a nitrogen flow rate of 30 mL·min^−1^.

#### 2.3.6. Scanning Electron Microscope (SEM) Testing

Micrographs of specimens, such as the bubbles and matrix, were analyzed by scanning electron microscope using JSM-6510 (JEOL, Tokyo, Japan). Conductive tapes were pasted on the surface of specimens, and a thin gold layer was sputtered for 80 s to maintain the electronic conductivity. SEM tests were conducted under the condition of high vacuum and at an acceleration voltage of 15 kV.

## 3. Results and Discussion

### 3.1. Distribution of Chopped Carbon Fibers in Resin Glue

**Figure 1:**
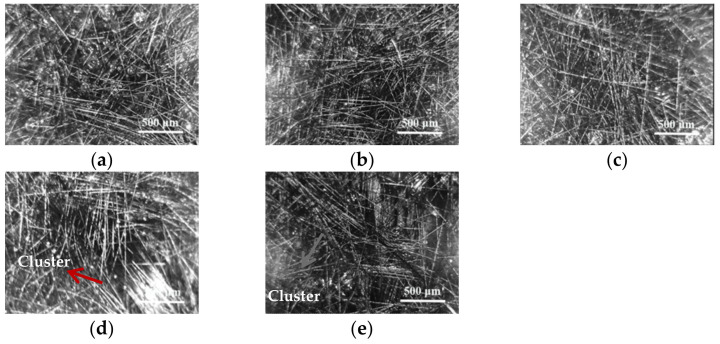
The distribution of 4.00 phr chopped carbon fibers with length of 2.0 mm (**a**), 4.0 mm (**b**), 6.0 mm (**c**), 8.0 mm (**d**), and 10.0 mm (**e**) in resin glue.

The length and content of chopped carbon fibers are both important factors, which affect the mechanical properties of composite material. The distribution of chopped carbon fibers in resin glue was observed by A005+ microscope (Weichuangjie Testing Instrument Co., Ltd., Shenzhen, China). According to previous researches, the length of chopped carbon fibers in composite materials of resin matrix was from 2.0 to 10.0 mm [12,13,14]. Therefore, five lengths of chopped carbon fibers were selected as various lengths of chopped carbon fibers in the study. The distribution of 4.00 phr chopped carbon fibers in length of 2.0, 4.0, 6.0, 8.0, and 10.0 mm in resin glue is shown in Figure 1. In Figure 1a–c, chopped carbon fibers in length of 2.0, 4.0, and 6.0 mm distribute homogenously in resin glue, which is favorable to enhance mechanical properties of solid composite material. However, Figure 1d,e indicate that chopped carbon fibers gather, form clusters, and distribute orientally in resin glue as the length of chopped carbon fibers changing up to 8.0 mm, which might be adverse to mechanical property of solid composite materials. It is considered that the longer the fiber length, the better mechanical properties of composite materials [15]. Therefore, 6.0 mm length of chopped carbon fibers was set as the reinforced chopped carbon fiber in the study.

**Figure 2:**
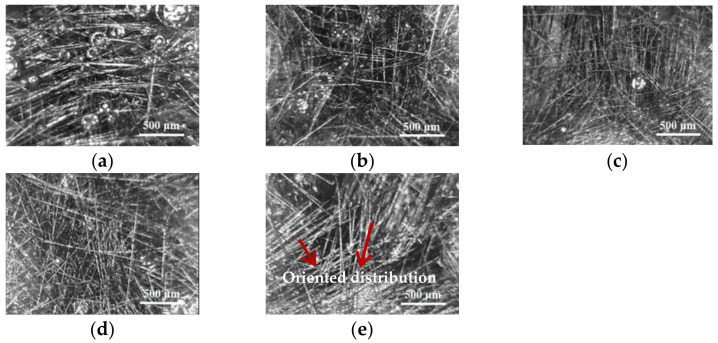
The distribution of 6.0 mm chopped carbon fibers with content of 1.00 phr (**a**), 2.00 phr (**b**), 3.00 phr (**c**), 4.00 phr (**d**), and 5.00 phr (**e**) in resin glue.

Distribution of different contents of 6.0 mm chopped carbon fibers in resin glue is shown in Figure 2. Figure 2 indicates that chopped carbon fibers interweave gradually with each other, and distribute homogenously in resin glue as the content of chopped carbon fibers changes from 1.00 to 4.00 phr. However, as the content of chopped carbon fibers is up to 5.00 phr, chopped carbon fibers cluster together and distribute orientally, which impinge on the mechanical properties of composite material. In order to explore the effect of different contents of chopped carbon fibers on CCFR-LDUPR composite, five contents of 6.0 mm chopped carbon fibers were selected in the research, which was 1.00, 2.00, 3.00, 4.00, and 5.00 phr respectively.

### 3.2. Viscosity of Resin Glue with Different Contents of Chopped Carbon Fibers

**Figure 3:**
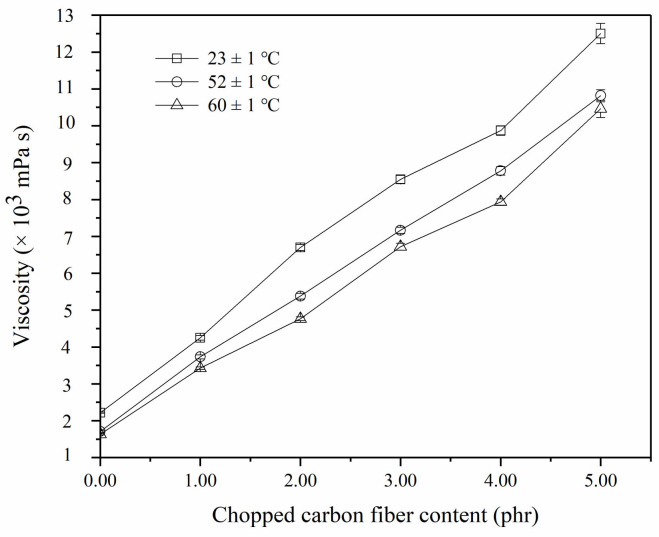
Viscosity changes of resin glue with different content of 6.0 mm chopped carbon fibers.

Although high addition of chopped carbon fibers improved the mechanical properties of this low-density composite, it significantly increased the viscosity of resin glue, which was adverse to bubbles forming and bubbles distribution resulting in a poor foaming resin matrix. Therefore, chopped carbon fibers were restricted to a certain amount. In the study, a high content of chopped carbon fibers in the composite material was explored. Viscosity changes of resin glue in the presence of 1.00, 2.00, 3.00, 4.00, and 5.00 phr, 6.0 mm chopped carbon fibers were detected at temperatures of 23 ± 1 °C, 52 ± 1 °C, and 60 ± 1 °C. Corresponding results are illustrated in Figure 3.

Viscosity of the resin glue increases from 4250 ± 60 to 9870 ± 130 mPa·s as the content of 6.0 mm chopped carbon fibers increasing from 1.00 to 4.00 phr at temperature of 23 ± 1 °C is shown in Figure 3. An obvious change of viscosity occurs during the range between 4.00 phr chopped carbon fibers and 5.00 phr chopped carbon fibers, and the viscosity of resin glue reaches up to 12,450 ± 275 mPa·s. It is deduced that chopped carbon fibers cluster seriously in resin glue, leading to higher flow retardance and even a drastic change of viscosity. This analysis coincides with the result of clustered chopped carbon fibers observed by A005+ microscope.

As shown in Figure 3, the viscosity of the resin glue in the presence of 5.00 phr chopped carbon fibers is 10,815 ± 165 mPa·s at a temperature of 52 ± 1 °C, which is 1635 mPa·s lower than that at a temperature of 23 ± 1 °C. The viscosity of resin glue in the presence of chopped carbon fibers decreases on a small scale as temperatures rise to 60 ± 1 °C. The viscosity of the resin glue in the presence of 5.00 phr chopped carbon fibers is 10,460 ± 235 mPa·s at a temperature of 60 ± 1 °C, which is 355 mPa·s lower than that at temperature of 52 ± 1 °C. It is revealed that the effect of temperature on the viscosity of the resin glue in the presence of chopped carbon fibers became weaker as the temperature rises.

### 3.3. Adjustment of Gel Time of Resin Glue

From 52.0 to 60.0 °C, NH_4_HCO_3_ showed a decomposition rate of 14~20 mL·g^−1^·min^−1^ [4]. Gel time of unsaturated polyester resin usually lasted 4 min [16,17]. Therefore, 56~80 mL·g^−1^ of gas could be produced by NH_4_HCO_3_ decomposition in 4 min at different temperatures ranging from 52.0 to 60.0 °C. Different apparent densities of low-density unsaturated polyester resin samples were calculated and were in the range of 0.41~0.50 g·cm^−3^ with the presence of 2.00 phr NH_4_HCO_3_ at temperature from 52.0 to 60.0 °C. These calculated values of apparent density matched those of low-density material in References of [5,18,19], which were between 0.30 g·cm^−3^ and 1.00 g·cm^−3^. Therefore, 52.0~60.0 °C was set as the curing temperature for CCFR-LDUPR samples preparation.

In a commercial process, gel time of a resin glue was usually controlled between 23 and 35 min [20,21,22]. As reported previously in the literature, gel time was adjusted utilizing initiator of tert-butylperoxy benzoate (TBPB, AkzoNobel, Shanghai, China) alone for low-density unsaturated polyester resin or low-density unsaturated polyester resin composite materials preparation to control the polymerization to be slow enough to maintain the homogeneous distribution of bubbles, and low-density unsaturated polyester resin or its composite was treated from 70 to 84 °C [4,5]. In the study, synergistic effects of initiator methyl ethyl ketone peroxide (MEKP-II) and accelerator cobalt naphthenate were put forward and explored. It included the adjustment of gel time and a lower curing temperature from 52.0 to 60.0 °C for CCFR-LDUPR sample preparation through the novel synergistic action. From the perspective of mechanical property and the microstructure of bubbles distribution, the facile curing and foaming process at a low temperature had achieved similar effects to that of curing and foaming process with an initiator alone.

In the presence of an initiator, methyl ethyl ketone peroxide (MEKP-II) of 2.00 phr, different gel time of resin glue in different ratios of accelerator to initiator at temperature of 52.0 °C is shown in Table 1. At a temperature of 52.0 °C, the gel time of the resin glue is 5.2 ± 0.2 min or 16.4 ± 0.5 min in Table 1 as for 1:5 or 1:10 ratio of accelerator to initiator, which represents 0.40 or 0.20 phr cobalt naphthenate. In the case, the gel time of the resin glue is out of the proper gel time of 23~35 min. However, as for 1:15 ratio of accelerator to initiator, a proper gel time of resin glue, which is 26.5 ± 0.3 min, was obtained and shown in Table 2. Therefore, the ratio of accelerator to initiator was defined as 1:15.

**Figure 4:**
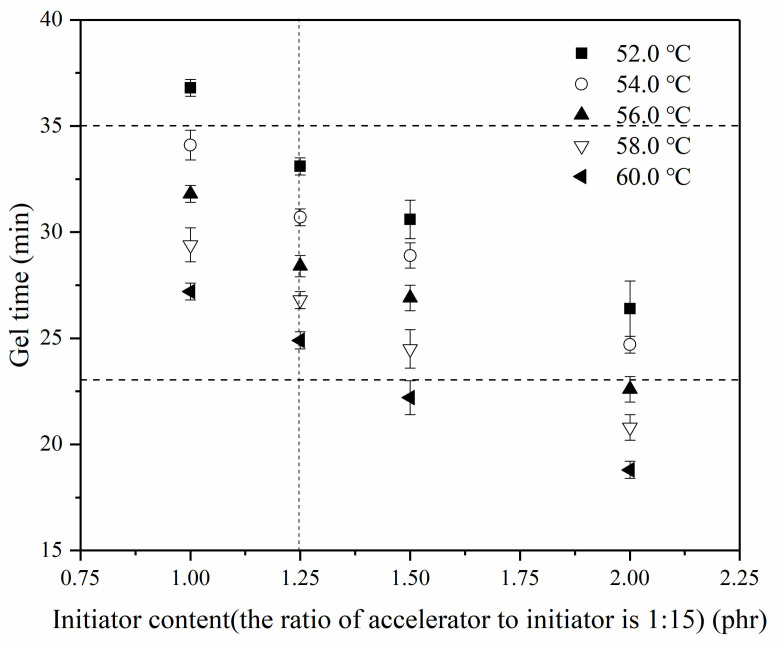
Gel time changes of the resin glue with various contents of initiator and accelerator at a temperature from 52.0 to 60.0 °C.

In accordance with the fixed ratio of accelerator to initiator, the gel time of resin glue was adjusted by changing the content of initiator and experimental results are listed in Figure 4. Figure 4 illustrates that from 52 to 60 °C, gel time of resin glue is between 33.1 ± 0.4 min and 24.9 ± 0.4 min for 0.08 phr accelerator together with 1.25 phr initiator, where the ratio of accelerator to initiator retains 1:15. Therefore, CCFR-LDUPR composite samples could be prepared under the condition of the proper gel time range.

### 3.4. Orthogonal Experimental Design

In order to explore the synergistic effects of methyl ethyl ketone peroxide (MEKP-II) and accelerators of cobalt naphthenate at a low temperature from 52.0 to 60.0 °C, an orthogonal experiment was designed for CCFR-LDUPR composite preparation. Compressive strength and apparent density of CCFR-LDUPR composite samples were detected. Based on these results, optimal parameters of CCFR-LDUPR composite sample preparation were obtained.

Mechanical properties of CCFR-LDUPR composite samples were estimated by these factors, such as length, the content of chopped carbon fiber, the content of foaming agent NH_4_HCO_3_, the initiator methyl ethyl ketone peroxide (MEKP-II), the accelerator cobalt naphthenate, and the curing temperature. According to References [6,12,23], and the results of microscope observation, together with the viscosity detection, the addition of 6.0 mm chopped carbon fibers was set from 1.00 to 5.00 phr in intervals of 1.00 phr. The filling amount of foaming agent was selected from 1.00 to 3.00 phr in intervals of 0.50 phr according to previous researches 5 and 11. Initiator methyl ethyl ketone peroxide (MEKP-II) and accelerator cobalt naphthenate were set as 1.25 and 0.08 phr, respectively, and the curing temperature was from 52.0 to 60.0 °C via gel time tests of resin glue in the presence of chopped carbon fiber. Therefore, three factors of the orthogonal experiment, such as curing temperature, filling amount of foaming agent, and addition of 6.0 mm chopped carbon fibers, as well as the different five levers of these factors, are list in Table 3.

The orthogonal experiment is described as L_25_(5^3^), where L is code name, 3 represents the number of factors, 5 indicates levels of each factor, and 25 represents the serial number of samples. Results of the L_25_(5^3^) orthogonal experiment are shown in Table 4.

### 3.5. Orthogonal Analysis of CCFR-LDUPR Composite Samples

Effects of the three factors on the density (*ρ*), compressive strength (*P*), and specific compressive strength (*P_s_*) of CCFR-LDUPR composite specimens were analyzed. *k*_1_, *k*_2_, and *k*_3_ is the mean of *ρ*, *P* and *P_s_* under a level of a certain factor, respectively. *R*_1_, *R*_2_, and *R*_3_ is the range corresponding to *k*_1_, *k*_2_, and to *k*_3_, respectively. Calculated values of *k* and *R* are list in Table 5.

CCFR-LDUPR is a lightweight reinforced composite material. In addition to apparent density *ρ* and compressive strength *P*, specific compressive strength *P_s_* is the comprehensive physical index for lightweight reinforced materials. It is revealed in Table 5 that three factors in descending order of importance for the range of specific compressive strength (*R*_3_) are the content of chopped carbon fibers (C), content of NH_4_HCO_3_ (B), and curing temperature (A). The major factor for *R*_3_ is C with a maximum range of 12.76 MPa·g^−1^·cm^3^, while the minor factor for *R*_3_ is A with a minimum range of 1.50 MPa·g^−1^·cm^3^. Therefore, the content of chopped carbon fiber (factor C) was the critical factor for properties of CCFR-LDUPR composite samples.

Effects of A, B, and C, three factors on properties of *ρ* and *P* and *P_s_* of the composite material, can also be discussed by the change of *k*. As shown in Table 5, *k_C_*_1_, which is the effect of chopped carbon fibers content (C) on *ρ*, shows an upward trend with the increase of chopped carbon fibers content. With the increase of chopped carbon fibers content, there was a more steric hindrance and even limited space for bubbles growth, diffusion and distribution. The fewer bubbles there are, the higher the apparent density for the CCFR-LDUPR sample is. *k_C_*_2_, which is the effect of factor C on compressive strength *P*, shows an upward trend with the increase of chopped carbon fibers content, indicating that the reinforcement of chopped carbon fibers to low-density unsaturated polyester resin increased with the increase of chopped carbon fiber content.

*k_C_*_3_, which is the effect of factor C on specific compressive strength *P_s_*, reaches up to the highest value of 45.19 MPa·g^−1^·cm^3^ in the presence of 4.00 phr chopped carbon fibers. However, *P_s_* decreases to 41.63 MPa·g^−1^·cm^3^ in the presence of 5.00 phr chopped carbon fibers. It is illustrated that with the change of chopped carbon fibers from 1.00 to 4.00 phr, chopped carbon fibers could homogenously distribute in resin glue and could later resolve the external force acted on solid composite samples. Nevertheless, there are obvious clusters and inhomogeneous distribution (including orientated distribution) of chopped carbon fibers in resin glue, which were adverse to resolve external force of the cured sample for the CCFR-LDUPR sample in the presence of 5.00 phr chopped carbon fibers. It is deduced that under the condition, growth, diffusion and distribution of bubbles were influenced by the amounts of chopped carbon fibers, resulting in the increase of density and the decrease of mechanical properties of cured CCFR-LDUPR sample in the presence of 5.00 phr chopped carbon fibers.

In Table 5, *k_B_*_1_ (which is the effect of foaming agent content (factor B) on apparent density *ρ*), and *k_B_*_2_ (which is the effect of foaming agent content (factor B) on compressive strength *P*) decrease with the increase of foaming agent content (B). It is considered that with the increase of foaming agent content, more bubbles formed in the composite samples and volume expansion occurred, which resulted in the decrease in apparent density. Therefore, with the volume expansion of the sample, the amount of chopped carbon fibers in a cubic volume of sample were turned down, and the compressive strength of CCFR-LDUPR composite samples decreased.

It is shown that in the presence of 2.00 phr NH_4_HCO_3_, the value of *k_B_*_3_ (which is the effect of factor B on specific compressive strength *P_s_*) reaches up to the highest one of 42.21 MPa·g^−1^·cm^3^. With the increase of NH_4_HCO_3_ from 1.00 to 2.00 phr, bubbles approached a saturated distribution in the resin glue. However, supersaturated bubbles squeezed in resin glue in the presence of 3.00 phr NH_4_HCO_3_. In the case, bubbles were compressed or destroyed, preventing normal formation and homogeneous distribution of bubbles, and reducing the compressive strength of the specimen.

In Table 5, *k_A_*_1_, which is the effect of curing temperature (factor A) on apparent density *ρ*, decreases with the increase of curing temperature. It is deduced that as the curing temperature rises, the amount of gas released by NH_4_HCO_3_ per unit time increased, resulting in an increase of the bubbles per unit volume for the CCFR-LDUPR composite sample. Therefore, the apparent density of CCFR-LDUPR composite samples decreased. As the temperature rose from 52.0 to 60.0 °C, *k_A_*_2_, which is the effect of curing temperature on the compressive strength, there were slight changes from 24.58 to 23.78 MPa. Meanwhile, the effect of factor A on specific compressive strength (*k_A_*_3_) reaches its highest value of 39.58 MPa·g^−1^·cm^3^ at the curing temperature of 58.0 °C.

Based on the above analysis, a sample was obtained in accordance with A_4_, B_3_, and C_4_ parameters, and was described as A_4_B_3_C_4_ sample. Theoretically, A_4_B_3_C_4_ sample performed optimal mechanical properties at the 4th level of factor A (at the preparation temperature of 58.0 °C), the 3rd level of factor B (in the presence of 2.00 phr NH_4_HCO_3_), and the 4th level of factor C (in the presence of 4.00 phr chopped carbon fibers). However, A_4_B_3_C_4_ sample, which presented optimal mechanical properties, was not included in 25 orthogonal experimental samples listed in Table 4. Therefore, it is necessary to verify the mechanical properties of A_4_B_3_C_4_ sample and compare them with those of sample of 8, which exhibited the highest mechanical property in 25 orthogonal experimental samples in Table 4. Corresponding results are shown in Table 6. 

Table 6 shows that the specific compressive strength of A_4_B_3_C_4_ sample reaches 53.56 ± 0.83 MPa·g^−1^·cm^3^ and is higher than that of sample of 8 (the highest one in orthogonal experiment listed in Table 4). It is verified that indexes of A_4_B_3_C_4_ sample were the optimal parameters for CCFR-LDUPR composite sample preparation, which were at a curing temperature of 58.0 °C, the content of NH_4_HCO_3_ of 2.00 phr, and in the presence of 4.00 phr chopped carbon fiber.

### 3.6. The Synergistic Effects of the Initiator and the Accelerator

In order to explore the synergistic effects caused by methyl ethyl ketone peroxide (MEKP-II) and accelerator of cobalt naphthenate in a low temperature range from 52 to 60 °C, an accelerator-free sample was designed and was described as (A_4_B_3_C_4_)^#^ sample. The curing temperature, the addition of a foaming agent, content of initiator, and the percent of chopped carbon fiber of (A_4_B_3_C_4_)^#^ sample were the same as those of A_4_B_3_C_4_ sample. Under the condition of similar gel time, apparent density and specific compressive strength of two samples are listed in Table 7 and compared.

In Table 7, the apparent density increases by 0.07 g·cm^−3^, the specific compressive strength decreases by 4.29 MPa·g^−1^·cm^3^, and the curing time (the time from the gel to the complete curing of the resin) is prolonged by 7.6 min for the (A_4_B_3_C_4_)^#^ sample. It is deduced that cobalt naphthenate accelerated the curing process of the A_4_B_3_C_4_ sample, and that the curing time was shortened. During the curing process of (A_4_B_3_C_4_)^#^, bubbles caused by NH_4_HCO_3_ decomposition might escape easily due to a longer curing time. As a result, apparent density increased and specific compressive strength decreased for the (A_4_B_3_C_4_)^#^ sample through the slower curing process.

**Figure 5:**
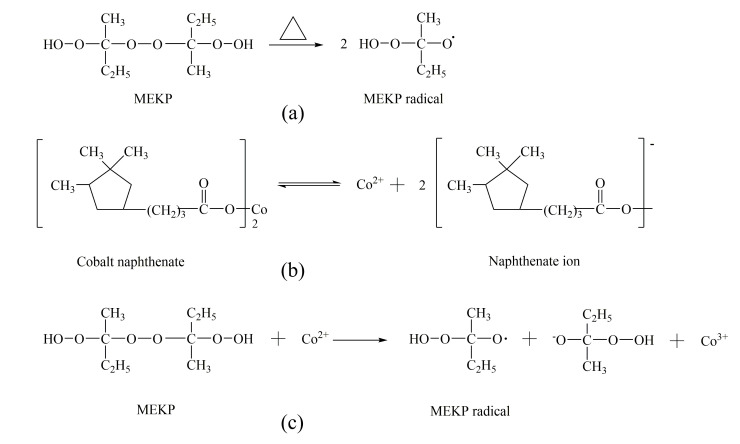
Formation diagram of different radicals: (**a**) free radicals generating without an accelerator; (**b**) the decomposition of cobalt naphthenate; (**c**) free radicals generated under duel synergistic effects of methyl ethyl ketone peroxide (MEKP-II) and cobalt naphthenate.

In Table 7, it is illustrated that under synergistic effects of the initiator and the accelerator, the curing process of the A_4_B_3_C_4_ sample is accelerated. However, the compressive strength of A_4_B_3_C_4_ sample is only 0.95 MPa lower than that of (A_4_B_3_C_4_)^#^ sample, which was without an accelerator. It is deduced that besides free radicals caused by methyl ethyl ketone peroxide (MEKP-II) (shown in Figure 5a), free radicals were also produced by the reaction between Co^2+^ and methyl ethyl ketone peroxide (MEKP-II) (shown in Figure 5b,c). Therefore, dominant free radical concentrations accelerated the curing process of the A_4_B_3_C_4_ sample and was favorable to the formation of a three-dimensional network of cured resin.

The curing process of resin was accelerated by the synergistic action of initiator and accelerator for CCFR-LDUPR composite samples. However, the facile polymerization would impinge on the mechanic properties of CCFR-LDUPR composite samples. Chopped carbon fibers, as a reinforced material, changed the disadvantage and reassembled the microstructure of the CCFR-LDUPR composite sample, resulting in the maintenance of performances. Therefore, under the synergistic action of initiator and accelerator, the excellent reinforcement of chopped carbon fibers was exhibited in the facile manufacture of CCFR-LDUPR composite samples. In that case, the compressive strength of the A_4_B_3_C_4_ sample was similar to that of the (A_4_B_3_C_4_)^#^ sample, which was without an accelerator and had a slower polymerization. It is unambiguous that the duel synergistic effects of an initiator and an accelerator had a significant effect on the facile and efficient polymerization of the unsaturated polyester resin. 

### 3.7. Non-Isothermal Differential Scanning Calorimetry Test Analysis

**Figure 6:**
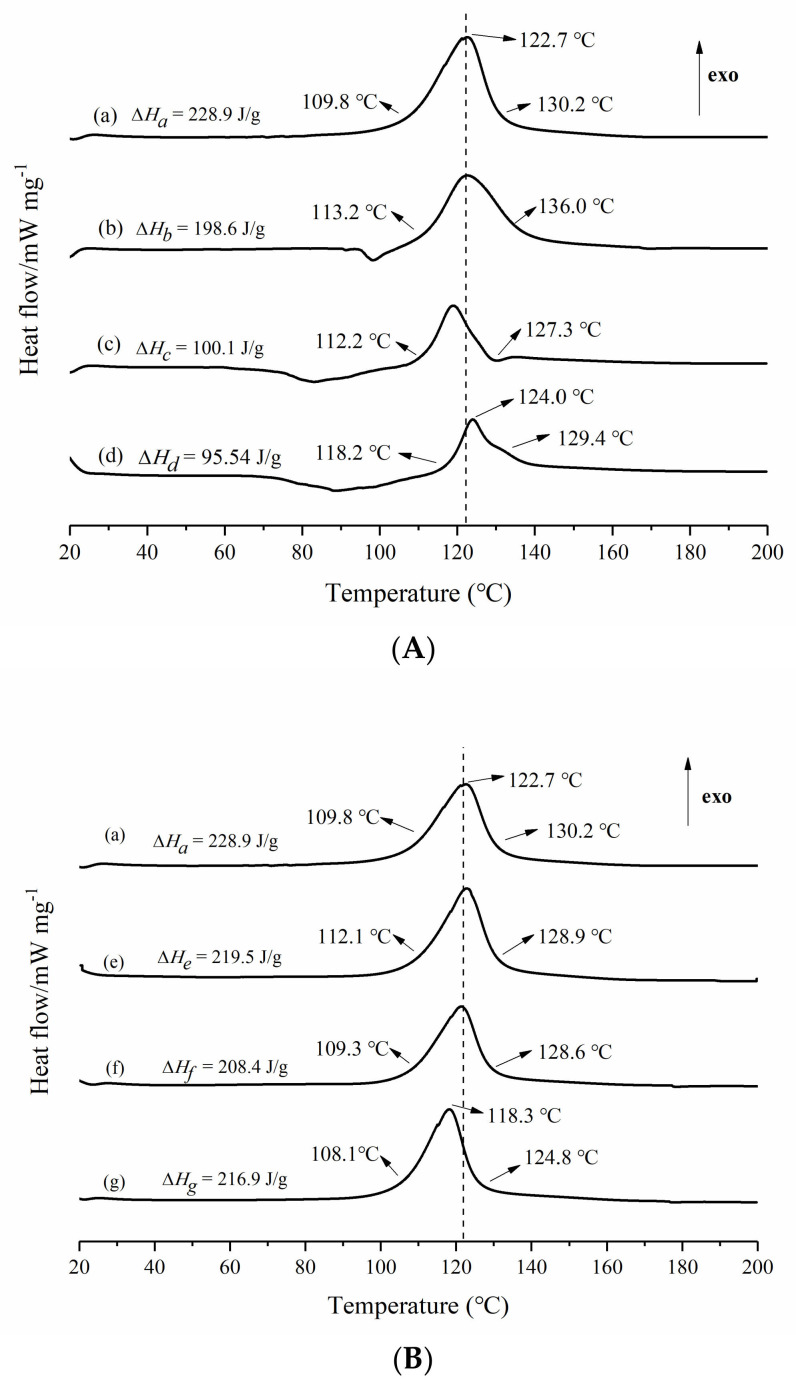

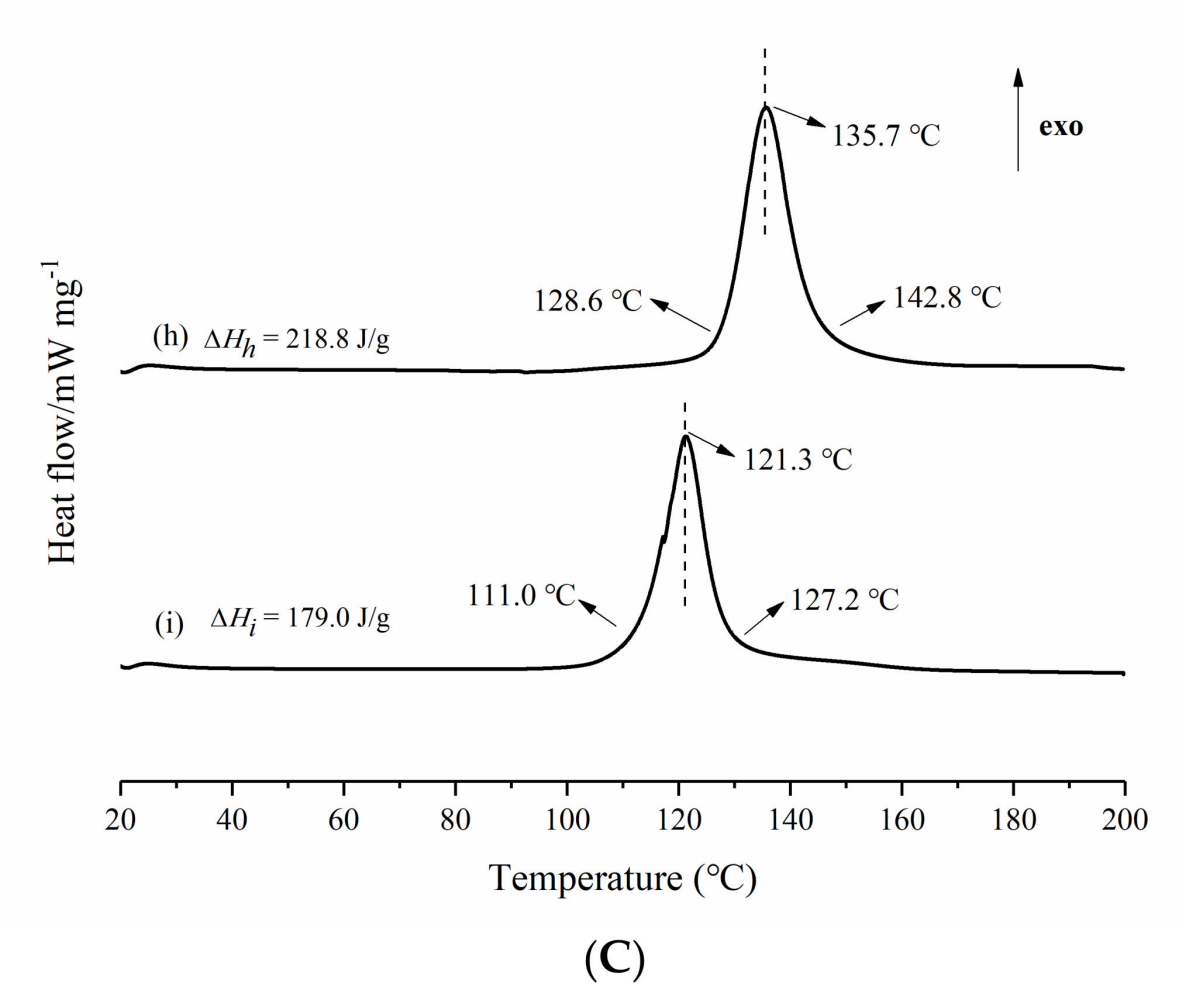
Non-isothermal differential scanning calorimetry curves of (**A**) wherein (a) pure UPR, (b) UPR + 1.00 phr NH_4_HCO_3_, (c) UPR + 2.00 phr NH_4_HCO_3_, (d) UPR + 3.00 phr NH_4_HCO_3_; (**B**) wherein (a) pure UPR, (e) UPR + 1.00 phr chopped carbon fibers, (f) UPR + 3.00 phr chopped carbon fibers, (g) UPR + 5.00 phr chopped carbon fibers; (**C**) wherein (h) UPR (accelerator-free) + 2.00 phr NH_4_HCO_3_ + 4.00 phr chopped carbon fibers, (i) UPR + 2.00 phr NH_4_HCO_3_ + 4.00 phr chopped carbon fibers.

In order to explore the effects of NH_4_HCO_3_, chopped carbon fibers, and the synergistic effects of an accelerator and initiator on the thermodynamic performances of unsaturated polyester resin during the low-temperature rapid curing process. Non-isothermal DSC experiments of resin glue in curing process were carried out and are shown in Figure 6.

The effects of NH_4_HCO_3_ on the thermodynamic performances of unsaturated polyester resin in the curing process are illustrated in Figure 6A. Figure 6A indicates that the curing exothermic heat of unsaturated polyester resin gradually decreases from 228.9 J/g (Δ*H_a_*) to 95.54 J/g (Δ*H_d_*) for the sample without NH_4_HCO_3_ and for the sample in the presence of 3.00 phr NH_4_HCO_3_. It is attributed to the decomposition and heat absorption of NH_4_HCO_3_ in an unsaturated polyester resin curing process of the sample, which led to the decrease of Δ*H*. In Figure 6A, the exothermic peak temperatures of the (a) curve, (b) curve, (c) curve, and the (d) curve are all at about 122 °C. However, the initial exothermic temperature changes from 109.8 to 118.2 °C, which was caused by the retardance of aqua generating during the decomposition of NH_4_HCO_3_ in the unsaturated polyester resin curing process. Furthermore, the peak width changes from 20.4 to 11.2 °C for four curves. It is considered that NH_4_HCO_3_ neutralized the residual acid (which was a retarder for polyester cross-linking) in unsaturated polyester resin glue and accelerated the polymerization of unsaturated polyester resin. Additionally, endothermic decomposition of NH_4_HCO_3_ accelerated the curing process of unsaturated polyester resin, resulting in the peak width of exothermic curve narrowing.

The effects of chopped carbon fibers on the thermodynamic performances of the unsaturated polyester resin curing process are exhibited in Figure 6B. As shown in Figure 6B, all of the initial exothermic temperatures are about 110 °C for the (a) curve, (e) curve, (f) curve, and (g) curve, and their exothermic peaks are nearly 120 °C. With the increase of chopped carbon fibers from 0 to 5.00 phr, the curing exothermic heat of unsaturated polyester resin decreases from 228.9 J/g (Δ*H_a_*) to 216.9 J/g (Δ*H_g_*). This is because the percentage of unsaturated polyester resin reduced in contrast with the increase in chopped carbon fibers content, resulting in a decrease in Δ*H*.

The synergistic effects of accelerator and initiator on thermodynamic performances of UPR in low-temperature facile preparation of CCFR-LDUPR is shown in Figure 6C. In Figure 6C, it is clear that the initial exothermic temperature of (i) the curve under synergistic effects of methyl ethyl ketone peroxide (MEKP-II) and cobalt naphthenate, is lower than 17.6 °C, compared with that of the (h) curve only in the presence of methyl ethyl ketone peroxide (MEKP-II). Furthermore, the exothermic peak temperature of the (i) curve is 121.3 °C, 17.6 °C lower than that of the (h) curve. Meanwhile, the curing exothermic heat of the (i) curve (Δ*H_i_*) is 39.8 J/g, lower than that of the (h) curve (Δ*H_h_*). It is concluded that the novel and critical synergistic effects of methyl ethyl ketone peroxide (MEKP-II) and cobalt naphthenate made a definitive contribution to the CCFR-LDUPR preparation.

### 3.8. Analysis of Scanning Electron Microscope (SEM) Test Results

**Figure 7:**
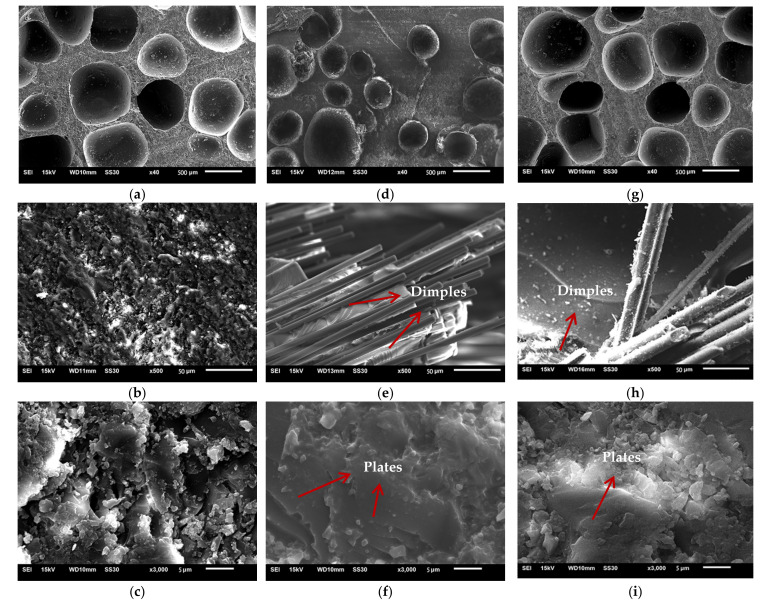
Scanning electron microscope micrographs of cured low-density unsaturated polyester resin sample (**a**–**c**), cured CCFR-LDUPR sample in presence of 4.00 phr chopped carbon fibers (**d**–**f**), and cured CGFR-LDUPR sample in the presence of 4.00 phr CGFs (**g**–**i**).

Micrographs of low-density unsaturated polyester resin, CCFR-LDUPR, and CGFR-LDUPR composite samples were observed by SEM and are shown in Figure 7.

Figure 7a shows that bubbles in the diameter of 600 μm distribute homogenously in the resin matrix of the low-density unsaturated polyester resin sample. As for the micrograph, the bubble area is 3.39 mm^2^, the area of micrograph is 6.03 mm^2^, and the bubble’s area ratio is calculated to be 56%. However, bubbles become smaller mainly in a diameter of 400 μm and in an inhomogeneous distribution in the presence of 4.00 phr chopped carbon fibers in Figure 7d. In this case, the bubble’s area ratio is 37% for the 4.00 phr CCFR-LDUPR sample. As for the CGFR-LDUPR sample with the presence of 4.00 phr chopped glass fibers, bubbles in a diameter of 600 μm are homogeneously distributed in the sample (Figure 7g), with a bubble’s area of 51%. It is obvious that both samples present a similar microstructure for low-density unsaturated polyester resin samples and for the CGFR-LDUPR sample with 4.00 phr chopped carbon fibers, while both samples exhibit similar *P_s_* values, which is 27.82 ± 1.13 MPa·g^−1^·cm^3^ and 28.56 ± 0.97 MPa·g^−1^·cm^3^, respectively. On the other hand, the apparent density of the CCFR-LDUPR sample in the presence of 4.00 phr chopped carbon fibers is 0.58 ± 0.02 g·cm^−3^, and the specific compressive strength is 53.06 ± 2.46 MPa·g^−1^·cm^3^ due to its lower bubble’s area ratio of 37%.

Besides the bubble’s area ratio, as shown in Figure 7b, e, h, at a magnification of up to 500×, chopped carbon fibers stack flatly layer by layer and distribute in bunches in the resin matrix of the CCFR-LDUPR sample. Chopped glass fibers distribute irregularly and coexist in some layers in the resin matrix of the CGFR-LDUPR sample. Besides, microcracks and microvoids appear in the rough matrix of the low-density unsaturated polyester resin sample. It is exactly the flat stack of chopped carbon fibers producing layers of the plateau microstructure for the CCFR-LDUPR sample, which prevented microcracks and microvoids. The flat stack of chopped glass fibers and its irregular distribution made layers of the plateau microstructure and microcracks and microvoids appear together in the resin matrix of the CGFR-LDUPR sample. It is considered that the flat stack of chopped fibers and layers of plateau microstructure were favorable to decompose external forces.

**Figure 8:**
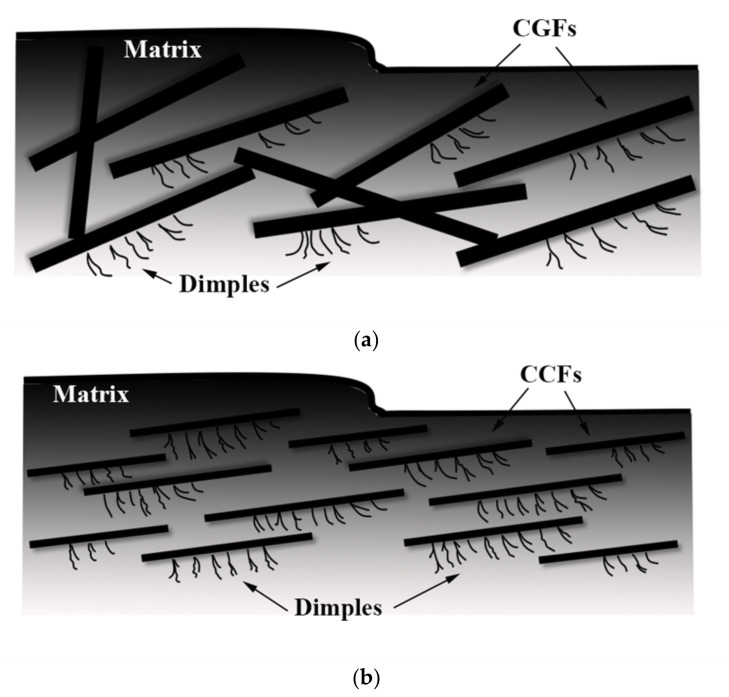
Schematic diagrams of “dimples” in (**a**) CGFR-LDUPR sample matrix and in (**b**) the CCFR-LDUPR sample matrix.

The microstructure of “dimples”, which adheres to chopped carbon fibers or chopped glass fibers, is obvious for both CCFR-LDUPR sample and the CGFR-LDUPR sample shown in Figure 7e, h. Branches of “dimples” are regular and with fine lines, and the schematic diagrams are illustrated in Figure 8a, b. However, there are no “dimples” in the resin matrix of the low-density unsaturated polyester resin sample. It is deduced that external forces were decomposed and transmitted along each branch of the “dimples” produced by chopped fibers, resulting in the improvement of mechanic performance of the matrix [5].

Figure 7c indicates that there are microcracks and microvoids in the rugged matrix of the low-density unsaturated polyester resin sample. However, a feature of “plate microstructure” is obvious in the micrograph of the CCFR-LDUPR sample with the presence of 4.00 phr chopped carbon fibers, where the resin matrix is flat and few microcracks or microvoids exist (see Figure 7f). This microstructure is different to that of th LDUPR sample. As for the CGFR-LDUPR sample with the presence of 4.00 phr chopped glass fibers, microstructural planeness of the resin matrix is better than that of the low-density unsaturated polyester resin sample, but rougher than that of the 4.00 phr CCFR-LDUPR sample (see Figure 7i). The microstructure of the CGFR-LDUPR sample, which is in the presence of 4.00 phr chopped glass fibers, has a tendency toward the feature of “plate microstructure” owing to the existence of chopped fibers.

**Figure 9:**
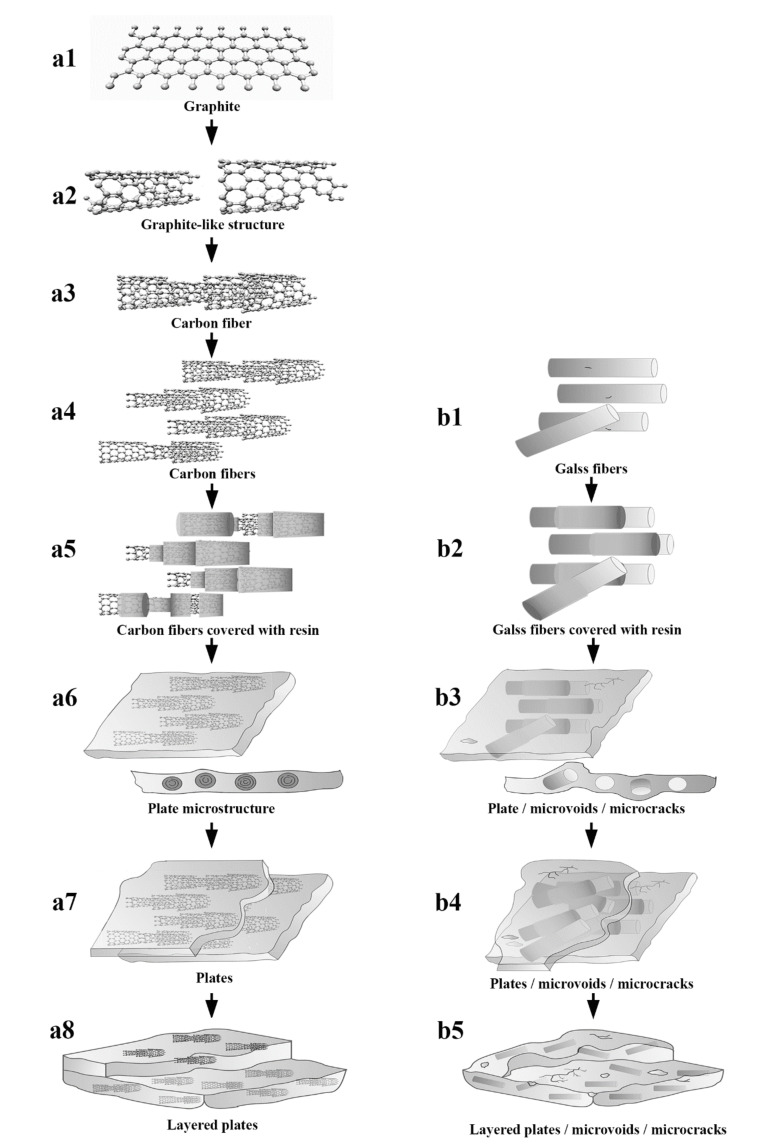
The formation mechanism diagrams of the matrix microstructure of CCFR-LDUPR and CGFR-LDUPR samples.

The microstructure of carbon fiber is irregular compared with that of graphite, which is shown in Figure 9(a1). A graphite-like structure of carbon fiber is similar to that of artificial graphite with layered graphene crystals and is in a disordered pack, which is illustrated in Figure 9(a2) [24,25]. Layers of carbon fiber are in a space of 3.40 ± 0.3 Å, and an irregularly layered graphite-like structure was connected by Van der Waals forces described as Figure 9(a3,a4) [26]. After the soaking of unsaturated polyester resin glue, an inner layer and an outer layer of the graphite-like structure of chopped carbon fibers were enwrapped by UPR glue (see Figure 9(a5)). Meanwhile, chopped carbon fibers were adhered to each other by resin glue.

The surface of chopped glass fibers was smooth but with some flaws (see Figure 9(b1)). After the soaking of UPR glue, resin glue covered the surface of chopped glass fibers illustrated in Figure 9(b2).

**Figure 10:**
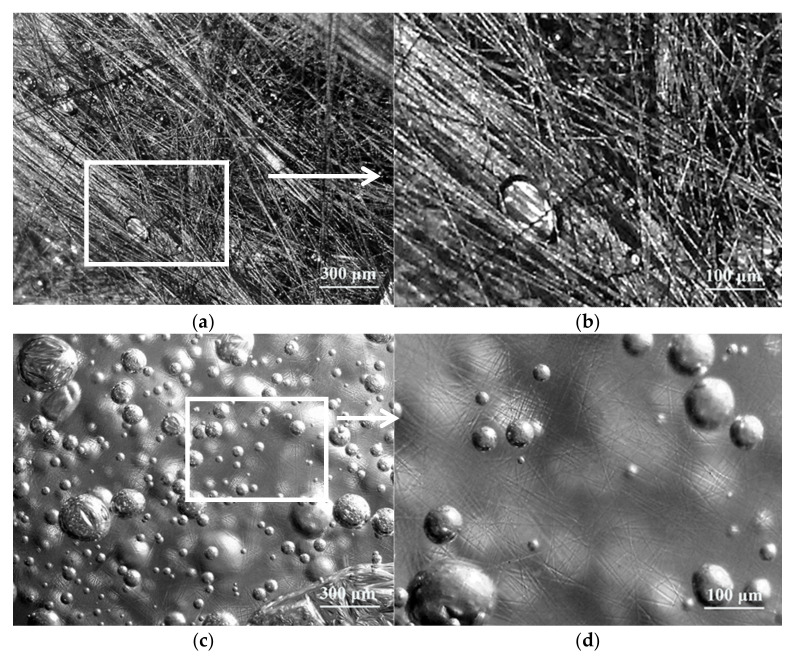
The distribution of 6.0 mm chopped carbon fibers with a content of 4.00 phr (**a**,**b**) and 6.0 mm chopped carbon fibers with a content of 4.00 phr (**c**,**d**) in resin glue.

The soakage and encasing of resin for chopped carbon fibers was different to that for chopped glass fibers. In the microstructure, a layered graphite-like structure of carbon fiber was enwrapped in unsaturated polyester resin glue from the surface to the inner layer, until chopped carbon fibers were completely soaked. As a result, irregular graphite-like structure of carbon fiber was covered with a smooth layer of resin glue. The amounts of soaked chopped carbon fiber were adhered to each other by resin glue, which became embryonic in the form of plates shown in Figure 10a, b. On the other hand, the surface of chopped glass fibers was covered with a smooth layer of resin glue and chopped glass fibers distributed in a random order in resin glue, as shown in Figure 10c,d.

**Figure 11:**
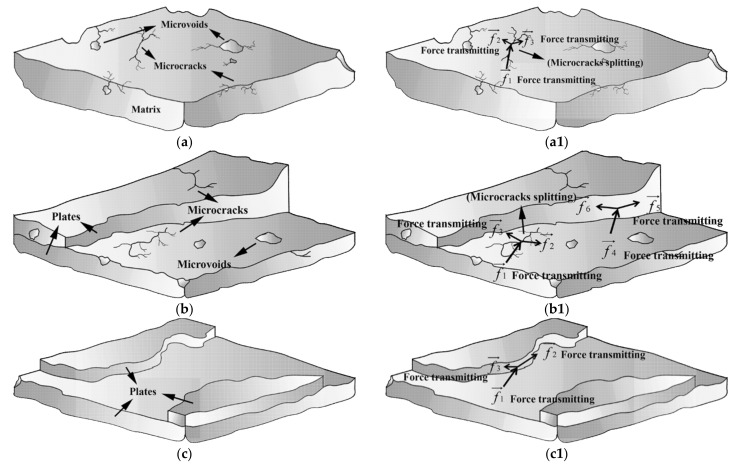
Microstructure schematic diagrams of (**a**) low-density unsaturated polyester resin sample matrix, (**b**) CGFR-LDUPR sample matrix, and (**c**) a CCFR-LDUPR sample matrix; the external force transmitting in (**a1**) LDUPR sample matrix, (**b1**) CGFR-LDUPR sample matrix, and (**c1**) CCFR-LDUPR sample matrix.

The density of carbon fiber was 1.8 g/cm^3^ and the density of glass fiber was 2.6 g/cm^3^, which meant that the density of carbon fiber was lower than that of glass fiber. Furthermore, the diameter of carbon fiber was 7 μm, which was smaller than that of glass fiber in a diameter of 13 μm. Considering the density and the diameter of carbon fiber and glass fiber, and in the case of the same length of 6.0 mm and the same addition of 4.00 phr for both chopped fibers, the filament number of chopped carbon fibers was much more than that of chopped glass fibers, as for unit mass of resin glue. It is proved by the distribution of chopped fibers in Figure 10b, d, where the filament number of chopped carbon fibers in a length of 6.0 mm is shown in Figure 11b is much more than that of chopped glass fibers shown in Figure 10d. Moreover, the filaments of chopped carbon fibers, which was in parallel and in a micro-plate, distribute in the resin glue as Figure 10b illustrated, while filaments of chopped glass fibers distribute in a random order in resin glue shown as Figure 10d is illustrated. It is revealed that the obvious distribution difference between filaments of chopped carbon fibers and filaments of chopped glass fibers.

During the later curing process of the CCFR-LDUPR sample, cured resin and soaked chopped carbon fibers, which were in parallel and in a micro-plate, which was a fabricated micro-scale plate illustrated in Figure 9(a7). Different to the CCFR-LDUPR sample, soaked chopped glass fibers were distributed in a random order in resin glue and it was difficult to form a large number of micro-scale plates. Besides, microcracks and microvoids were easy to form due to the irregular distribution of chopped glass fibers in resin glue. Meanwhile, a few of the micro-scale plates emerged for the CGFR-LDUPR sample. Microcracks and microvoids introduced internal forces in the resin matrix of the CGFR-LDUPR sample, which is shown in Figure 9(b4). Finally, layered micro-scale plates joined together and formed a specific “plate microstructure” of the CCFR-LDUPR sample, which is indicated in Figure 9(a8). On the other hand, the microstructure of the CGFR-LDUPR sample consisted of a small amount of “plate microstructure”, microcracks, and microvoids shown in Figure 9(b5).

With the magnification of 3000×, there are microcracks and microvoids in the matrix of the low-density unsaturated polyester resin sample (Figure 7c), which is regarded as a “body defect” for this kind of composite material microstructure. It is deduced that the “body defect” of a sample matrix was adverse to bear the external force because microcracks might diffuse and microvoids might break under the action of an external force, resulting in a decrease in mechanical properties.

However, a feature of the “plate microstructure” is the micrograph specialty of the CCFR-LDUPR sample with the presence of 4.00 phr chopped carbon fibers without microcracks and microvoids (Figure 7f). “Plate microstructure” packs up layer by layer but not in one piece as for the microstructure of the matrix. This specific microstructure is regarded as a “surface defect” of composite material. The microstructure schematic diagrams of three kinds of matrix are illustrated in Figure 11a–c, and the external force transmitting in three kinds of matrix are illustrated in Figure 11(a1–c1). It is deduced that chopped carbon fibers combined firmly with the resin matrix and prevented the cracks of the resin matrix, resulting in the formation of “plate microstructure” for the resin matrix. Thin carbon fiber in a diameter of about 7 μm was further favorable to prevent the crack of resin matrix [27,28]. It is considered that the “surface defect” of the sample matrix was favorable to bear the external force because the “plate microstructure” could carry out an external force as a whole and resolve the external force one plate by one plate (see Figure 11(c1)). Since there were no internal microcracks or microvoids, no microcracks diffuse and no microvoids break under the action of an external force. Therefore, the mechanical properties of the sample were improved in the case of “surface defect”.

“Surface defect” and “body defect” co-exist in the microstructure of the 4.00 phr CGFR-LDUPR sample, where “plate microstructure”, microcracks, and microvoids are visible in Figure 7i. It is deduced that mechanical properties of the sample were better than those of the LDUPR sample but poorer than those of the 4.00 phr CCFR-LDUPR sample. Consequently, the “body defect” formed in the resin matrix of low-density unsaturated polyester resin with no chopped fibers, “surface defect” and “body defect” co-existed in the presence of chopped glass fiber, while chopped carbon fiber produced a particular “surface defect” microstructure of composite material. Different defects resulted in different composite materials performing different mechanical properties.

Chopped carbon fiber caused a particular “surface defect” microstructure in the resin matrix, and a different content of chopped carbon fibers had different effects on the microstructural change of the CCFR-LDUPR samples.

**Figure 12:**
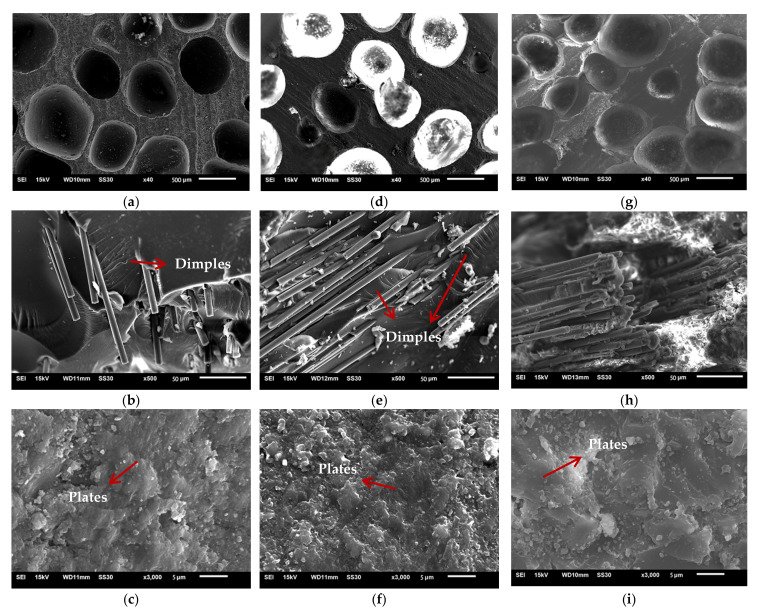
SEM micrographs of cured CCFR-LDUPR sample in the presence of 1.00 phr chopped carbon fibers (**a**–**c**), cured CCFR-LDUPR sample in the presence of 3.00 phr chopped carbon fibers (**d**–**f**), and cured CCFR-LDUPR sample in the presence of 5.00 phr chopped carbon fibers (**g**–**i**).

Micrographs of the CCFR-LDUPR samples in the presence of 1.00 phr chopped carbon fibers, 3.00 phr chopped carbon fibers, and of 5.00 phr chopped carbon fibers are represented in Figure 12. With the addition of 1.00 phr chopped carbon fibers or 3.00 phr chopped carbon fibers, the diameter of the bubble is mainly about 600 mm. Combining Figure 7 and Figure 12, it can be found that as the content of chopped carbon fibers reaches up to 4.00 phr and 5.00 phr, the coexistence of bubbles in diameter ranges from 400 to 600 mm occurred in CCFR-LDUPR samples (see Figure 12a,d,g). It is indicated that a low content of chopped carbon fibers had little effect on the formation of bubbles, while a higher content of chopped carbon fibers (more than 4.00 phr) restricted the space of bubbles foaming. Under this condition, bubbles became smaller, linked bubbles formed and larger bubbles, together with smaller bubbles, coexisted, which is visible in the 5.00 phr CCFR-LDUPR sample (see Figure 12g).

At a magnification of 500×, it can be found that, as the content of chopped carbon fibers increases from 1.00 to 5.00 phr, chopped carbon fibers in the resin matrix gradually change from a sparse distribution to a flat stack. Moreover, with the increase of chopped carbon fibers content, the amounts of “dimples” attaching to chopped carbon fibers in the CCFR-LDUPR sample matrix increase and they are regular with fine lines (see Figure 12b,e). As the chopped carbon fiber content reaches up to 5.00 phr, the amounts of “dimples” in the CCFR-LDUPR sample matrix decreases (see Figure 12h). The flat stack of chopped carbon fibers with the addition of 4.00 and 5.00 phr promoted the formation of a “plate microstructure” for the CCFR-LDUPR sample. With the presence of 4.00 phr chopped carbon fibers, “plate microstructure” is the most obvious and this result coincides with the optimal flat stack of chopped carbon fibers distribution in Figure 7e. However, with the presence of 5.00 phr, chopped carbon fibers were unevenly packed and resulted in the appearance of a rolling “plate microstructure”. 

As the content of chopped carbon fibers increased from 1.00 to 5.00 phr, the feature of “plate microstructure” of the matrix gradually becomes obvious (see Figure 12c,f,g), while the compressive strength of the corresponding CCFR-LDUPR sample is 19.71 ± 0.68 MPa and 25.82 ± 0.74 MPa, respectively. Combined with the micrograph of Figure 7f, the feature of “plate microstructure” of the matrix is the most significant, as the chopped carbon fiber content reaches up to 4.00 phr. In this case, the CCFR-LDUPR sample with the presence of 4.00 phr chopped carbon fibers presented the highest value of *P*, which was 31.24 ± 0.47 MPa, and the corresponding *P_s_* was 53.56 ± 0.83 MPa·g^−1^·cm^3^. 

It is confirmed that the specific “dimples”, “plate microstructure” and “surface defect” is essential for chopped carbon fibers to reinforce the mechanical properties of CCFR-LDUPR composite samples in light of the above analysis, which is rather different from the low-density unsaturated polyester resin sample and the CGFR-LDUPR composite sample.

## 4. Conclusions 

CCFR-LDUPR composite materials with lightweight and high mechanical properties were prepared at a low temperature from 52.0 to 60.0 °C through synergistic effects of methyl ethyl ketone peroxide (MEKP-II) and cobalt naphthenate, which are novel and are put forward in this study. Optimal preparation conditions were obtained through orthogonal experiments, for which the preparation temperature was at 58.0 °C, the content of NH_4_HCO_3_ was 2.00 phr, and the content of chopped carbon fibers was 4.00 phr. The CCFR-LDUPR composite sample presented its optimal properties, which was *ρ* was 0.58 ± 0.02 g·cm^−3^ and *P_s_* was 53.56 ± 0.83 MPa·g^−1^·cm^3^, which is 38.9% higher than that of CGFR-LDUPR composite materials.

The curing mechanism of CCFR-LDUPR was explored by a non-isothermal DSC experiment. The aqua generated during the decomposition of NH_4_HCO_3_ had retardance effects in the UPR curing process, while endothermic decomposition of NH_4_HCO_3_ accelerated the curing reaction. Synergistic effects of methyl ethyl ketone peroxide (MEKP-II) and cobalt naphthenate advanced the initial temperature and the peak temperature of an exothermic curve, which accelerated the curing progress of UPR. Microscopic analysis shows that with the presence of chopped carbon fibers, unique “dimples”, a “plate microstructure” and a “surface defect” fabricated the specific microstructure of the matrix of CCFR-LDUPR composite samples. The microstructure was different from the “body defect” microstructure of the pure resin matrix, and was also different from the microstructure of the CGFR-LDUPR sample with a coexistence of “surface defect” and “body defect”. It is considered that the specific “dimples”, “plate microstructure” and “surface defect” microstructures were favorable to resolve the external force, and were essential for chopped carbon fiber to improve the mechanical properties of CCFR-LDUPR composite samples.

## Figures and Tables

**Table 1:** Component of CCFR-LDUPR composite samples.

Component	Content (phr)
NH_4_HCO_3_	A
chopped carbon fiber	B
methyl ethyl ketone peroxide	C
cobalt naphthenate	D

**Table 2:** Gel time of resin glue with different ratios of accelerator to initiator.

Accelerator to Initiator	Gel Time (min)
1:5	5.2 ± 0.2
1:10	16.4 ± 0.5
1:15	26.5 ± 0.3

**Table 3:** Factors and levels of orthogonal experiment.

Factors	Level 1	Level 2	Level 3	Level 4	Level 5
Fabrication temperature (°C) (A)	52.0	54.0	56.0	58.0	60.0
NH_4_HCO_3_ content (phr) (B)	1.00	1.50	2.00	2.50	3.00
chopped carbon fibers content (phr) (C)	1.00	2.00	3.00	4.00	5.00

**Table 4:** Orthogonal design and results of CCFR-LDUPR composite samples.

Sample Serial Number	Fabrication Temperature (°C) (A)	NH_4_HCO_3_ Content (phr) (B)	CCF Content (phr) (C)	*ρ* (g·cm^−3^)	*P* (MPa)	*P_s_* (MPa·g^−1^·cm^3^)
1	52.0	1.00	1.00	0.74 ± 0.04	21.38 ± 1.78	28.73 ± 1.03
2	52.0	1.50	2.00	0.65 ± 0.03	25.03 ± 0.44	38.54 ± 0.98
3	52.0	2.00	3.00	0.59 ± 0.03	26.31 ± 1.47	44.58 ± 1.46
4	52.0	2.50	4.00	0.58 ± 0.02	25.86 ± 1.69	44.82 ± 1.36
5	52.0	3.00	5.00	0.60 ± 0.02	24.34 ± 1.25	40.54 ± 0.73
6	54.0	1.00	2.00	0.76 ± 0.04	21.29 ± 1.43	28.14 ± 1.29
7	54.0	1.50	3.00	0.67 ± 0.04	25.16 ± 1.79	37.72 ± 0.71
8	54.0	2.00	4.00	0.60 ± 0.02	31.63 ± 0.84	52.72 ± 0.35
9	54.0	2.50	5.00	0.68 ± 0.04	26.31 ± 0.82	38.54 ± 1.40
10	54.0	3.00	1.00	0.54 ± 0.02	19.25 ± 0.69	35.66 ± 0.79
11	56.0	1.00	3.00	0.75 ± 0.03	25.53 ± 0.88	33.91 ± 1.02
12	56.0	1.50	4.00	0.70 ± 0.04	29.77 ± 1.31	42.45 ± 1.54
13	56.0	2.00	5.00	0.65 ± 0.06	26.70 ± 0.68	41.37 ± 1.47
14	56.0	2.50	1.00	0.50 ± 0.02	19.96 ± 1.18	39.65 ± 0.59
15	56.0	3.00	2.00	0.57 ± 0.03	21.92 ± 0.76	38.72 ± 1.73
16	58.0	1.00	4.00	0.68 ± 0.02	29.92 ± 0.90	44.04 ± 0.93
17	58.0	1.50	5.00	0.61 ± 0.03	25.69 ± 0.95	42.37 ± 1.21
18	58.0	2.00	1.00	0.54 ± 0.02	19.78 ± 0.68	36.44 ± 0.88
19	58.0	2.50	2.00	0.58 ± 0.03	21.11 ± 1.02	36.60 ± 0.17
20	58.0	3.00	3.00	0.65 ± 0.03	24.61 ± 0.29	37.69 ± 1.00
21	60.0	1.00	5.00	0.64 ± 0.02	29.35 ± 0.95	45.64 ± 0.93
22	60.0	1.50	1.00	0.74 ± 0.02	19.06 ± 0.88	25.90 ± 1.56
23	60.0	2.00	2.00	0.61 ± 0.03	21.66 ± 0.62	35.71 ± 0.45
24	60.0	2.50	3.00	0.58 ± 0.02	25.06 ± 0.72	43.48 ± 1.20
25	60.0	3.00	4.00	0.56 ± 0.02	23.80 ± 0.71	42.51 ± 1.03

CCF means chopped carbon fiber.

**Table 5:** Analysis of orthogonal experiment.

Factors	Mean	Level 1	Level 2	Level 3	Level 4	Level 5	*R*_1_, *R*_2_ or *R*_3_ (*k_max_*−*k_min_*)
Fabrication temperature (A)	*k_A_*_1_ (g·cm^−3^)	0.63	0.65	0.63	0.61	0.62	0.04
	*k_A_*_2_ (MPa)	24.58	24.73	24.78	24.22	23.78	0.99
	*k_A_*_3_ (MPa·g^−1^·cm^3^)	38.90	38.08	39.04	39.58	38.08	1.50
Content of NH_4_HCO_3_ (B)	*k_B_*_1_ (g·cm^−3^)	0.72	0.67	0.60	0.58	0.58	0.13
	*k_B_*_2_ (MPa)	25.49	24.94	25.21	23.66	22.78	2.71
	*k_B_*_3_ (MPa·g^−1^·cm^3^)	35.64	37.08	42.21	40.56	39.01	6.57
Content of chopped carbon fibers (C)	*k_C_*_1_ (g·cm^−3^)	0.61	0.63	0.65	0.62	0.64	0.03
	*k_C_*_2_ (MPa)	19.89	22.20	25.33	28.20	26.48	8.31
	*k_C_*_3_ (MPa·g^−1^·cm^3^)	32.42	35.16	39.10	45.19	41.63	12.76

*k*_1_: the mean of *ρ* calculated from five values under one level for a single factor; *R*_1_: the range between *k*_1*max*_ and *k*_1*min*__,_ *k*_2_: the mean of *P* calculated from five values under one level for a single factor; *R*_2_: the range between *k*_2*max*_ and *k*_2*min*__,_ *k*_3_: the mean of *P_s_* calculated from five values under one level for a single factor; *R*_3_: the range between *k*_3*max*_ and *k*_3*min*_.

**Table 6:** The verification experiment of A_4_B_3_C_4_ sample and parameters comparison.

Sample	Fabrication Temperature A (°C)	NH_4_HCO_3_ Content B (phr)	CCF Content C (phr)	*ρ* (g·cm^−3^)	*P* (MPa)	*P_s_* (MPa·g^−1^·cm^3^)
A_4_B_3_C_4_	58.0	2.00	4.00	0.58 ± 0.02	31.24 ± 0.47	53.56 ± 0.83
8^#^	54.0	2.00	4.00	0.60 ± 0.02	31.63 ± 0.84	52.72 ± 0.35

**Table 7:** Parameter comparison between A_4_B_3_C_4_ and (A_4_B_3_C_4_)^#^.

Sample	Initiator Content (phr)	Accelerator Content (phr)	Gel Time (min)	Curing Time (min)	*ρ* (g·cm^−3^)	*P* (MPa)	*P_s_* (MPa·g^−1^·cm^3^)
A_4_B_3_C_4_	1.25	0.08	26.8 ± 0.4	30.6 ± 0.9	0.58 ± 0.02	31.24 ± 0.47	53.56 ± 0.83
(A_4_B_3_C_4_)^#^	2.50	-	27.6 ± 0.7	38.2 ± 0.8	0.65 ± 0.02	32.19 ± 1.28	49.27 ± 1.05

## Data Availability

Data sharing not applicable.

## Abbreviations

UPR	Unsaturated polyester resin
CCF	Chopped carbon fiber
CGF	Chopped glass fiber
LDUPR	Low-density unsaturated polyester resin
CFRP	Carbon fiber reinforced plastic
CCFR-LDUPR	Chopped carbon fiber reinforced low-density unsaturated polyester resin
CGFR-LDUPR	Chopped glass fiber reinforced low-density unsaturated polyester resin
MEKP-Ⅱ	Methyl ethyl ketone peroxide
phr	Parts per hundreds of resin
TBPB	Tert-butylperoxy benzoate
DSC	Differential scanning calorimetry
SEM	Scanning electron microscope
